# Identification of Prognostic Genes in Neuroblastoma in Children by Weighted Gene Coexpression Network Analysis

**DOI:** 10.1155/2021/9987990

**Published:** 2021-07-27

**Authors:** Jun Yang, Ying Zhang, Jiaying Zhou, Shaohua Wang

**Affiliations:** ^1^Department of Pediatrics, The University of Hong Kong-Shenzhen Hospital, Shenzhen, China; ^2^Department of Pediatrics, Women and Children Institute of Futian, University of South China, Shenzhen, China

## Abstract

**Background:**

Neuroblastoma is a malignant neuroendocrine tumor from the sympathetic nervous system, the most common extracranial tumor in children. Identifying potential prognostic markers of neuroblastoma can provide clues for early diagnosis, recurrence, and treatment.

**Methods:**

RNA sequence data and clinical features of 147 neuroblastomas were obtained from the TARGET (Therapeutically Applicable Research to Generate Effective Treatments project) database. Application weighted gene coexpression network analysis (WGCNA) was used to construct a free-scale gene coexpression network, to study the interrelationship between its potential modules and clinical features, and to identify hub genes in the module. We performed Lasso regression and Cox regression analyses to identify the three most important genes and develop a new prognostic model. Data from the GSE85047 cohort verified the predictive accuracy of the prognostic model.

**Results:**

14 coexpression modules were constructed using WGCNA. Brown coexpression modules were found to be significantly associated with disease survival status. Multivariate Cox analysis was performed on genes from univariate Cox regression and Lasso regression analyses using the Cox proportional hazards regression model. Finally, we constructed a three-gene prognostic model: risk score = (0.003812659*∗*CKB) + (−0.152376975*∗*expDST) + (0.032032815*∗*expDUT). The prognosis of samples in the high-risk group was significantly poorer than that of samples in the low-risk group (*P*=1.225*e* − 06). The risk model was also regarded as an independent predictor of prognosis (HR = 1.632; 95% CI = 1.391–1.934; *P* < 0.001).

**Conclusion:**

Our study constructed a neuroblastoma coexpressing gene module and identified a prognostic potential risk model for prognosis in neuroblastoma.

## 1. Introduction

Neuroblastoma is the most common extracranial tumor in children and is the most common tumor in infants and young children [1]. Nearly half of the neuroblastomas occur in infants and young children under 2 years of age. Neuroblastoma is a neuroendocrine tumor that can originate from any nerve ridge of the sympathetic nervous system. The most common site of development is the adrenal gland, but it can also occur in the neck, chest, abdomen, and pelvic nerve tissue. A small number of human tumors are known to spontaneously degenerate from undifferentiated malignant tumors to completely benign tumors. Neuroblastoma is one of them [2].

The current cause of neuroblastoma is unclear. There are a variety of clinical and biological factors including staging and age of diagnosis. MYCN amplification and overexpression in NB is an important evaluation index for the classification of malignant degree and rapid progress [3]. Some genetic susceptibility factors have been found to be associated with the pathogenesis of neuroblastoma. Familial neuroblastoma has been shown to be caused by somatic mutations in anaplastic lymphoma kinase (ALK). In addition, many molecular mutations have been found in neuroblastoma. Amplification mutations in the N-myc gene are also common in neuroblastoma. Its amplification type is bidirectionally distributed: 3–10 fold amplification at one extreme and 100–300 fold amplification at the other extreme. Amplification mutations in the MYCN gene are often associated with tumor spread [4].

Weighted Gene Coexpression Network Analysis (WGCNA) is an effective method to study the association between gene coexpression modules and complex phenotypes of cDNA microarrays or RNA sequencing data [5]. It is also widely used in many genomic studies of cancer pathogenesis, such as the identification of gene expression modules associated with oncogenic signaling pathways or clinical variables, including molecular subtypes, grading/staging, and survival outcomes [6].

In this study, we applied WGCNA to publicly available microarray data, identified coexpression subnetworks, and detected oncogenic modules related to patient survival. Our comprehensive analysis provides new insights into the genetic characteristics of neuroblastoma and provides a potential genetic marker for tumor diagnosis and treatment.

## 2. Materials and Methods

### 2.1. Gene Expression Data

The RNA sequence data and corresponding clinical information of neuroblastoma were obtained from the Therapeutically Applicable Research to Generate Effective Treatments project database (TARGET, https://ocg.cancer.gov/programs/target), containing 147 tumor patients' tissues. Both mRNA profile data and clinical characteristics of neuroblastoma are publicly available and in open access platforms. Therefore, approval by the local ethics committee was not needed.

### 2.2. Weighted Gene Coexpression Network Analysis

The WGCNA algorithm is used in R (https://www.r-project.org/) to identify coexpression modules. We used weighted gene coexpression network analysis (WGCNA) to analyze a comprehensive network that can describe patterns and gene expression profiles and to assess the importance of genes and their module members. We use the Pearson correlation between any two genes to evaluate the weighted coexpression relationship between the subjects of all datasets in the adjacency matrix. In order to measure whether two genes have similar expression patterns, thresholds are generally required to be screened, and those above the threshold are considered similar [7]. WGCNA analysis uses the correlation coefficient weighting value (*a*_*mn*_=|cor(*x*_*m*_, *x*_*n*_)|^*β*^)so that the connections between the genes in the network obey the scale-free networks. This algorithm is more biologically significant. The parameter *β* of the standard scale-free network is determined by the following criteria: (1) The resulting adjacency matrix approximates the scale-free topological feature according to the previously proposed model fitting index. (2) The model fit index for a perfect scale-free network is 1. Here, the beta value in both analyses is determined to be 5, which is the minimum required to make the model fit index above 0.9. When the degree of independence is 0.9, the appropriate power value is determined (power values range from 1 to 20). Once the power value is determined, the WGCNA algorithm continues the module construction. In this study, the soft threshold was set to *β* = 5 (no scale *R*^2^ = 0.9). Based on the weighted correlation coefficient of genes, genes are classified according to expression patterns, and genes with similar patterns are classified into one module. In this way, tens of thousands of genes can be divided into dozens of modules through gene expression patterns, which is a process of extracting and inducting information [8]. The adjacency between genes is calculated, the similarity between genes is calculated according to the adjacency, and then the coefficient of dissimilarity between genes is derived, and a systematic clustering tree between genes is obtained accordingly. A similarity measure was defined: TOM_*mn*_=(∑_*u*_*a*_*mu*_*a*_*un*_+*a*_*mn*_/(min(*k*_*m*_, *k*_*n*_)+1 − *a*_*mn*_)), where *k*_*m*_=∑_*u*_*a*_*mu*_ was the node connectivity. After the gene module is determined according to the dynamic shearing method, the eigenvector value of each module is calculated in turn, and then the module is clustered and analyzed, and the modules with close distance are merged into a new module. The correlation between the module eigengene and the phenotype (clinical features) was used to estimate the module-trait association, which allows for easy identification of expression groups (modules) that are highly correlated with the phenotype. For each expression profile, gene significance (GS) was calculated as the absolute value of the correlation between the expression profile and each trait; module membership (MM) was defined as the correlation of the expression profile to each module eigengene [9].

### 2.3. Gene Functional Enrichment Analysis

Gene functional enrichment analysis was applied to the differentially expressed genes and gene modules identified by WGCNA. Using the R software clusterProfiler package to perform GO enrichment and KEGG pathway analysis on the genes in the obtained pivot module, the false discovery rate (FDR) <0.05 was considered statistically significant [10].

### 2.4. Identification and Selection of Prognosis-Related Genes

The R package “survival” was applied to carry out univariate Cox regression analysis on the key modules to identify the prognostic genes, and Lasso regression was performed to further screen important key genes. Finally, based on the preliminary screening of the above key candidate genes, we built a multivariate Cox proportional hazard regression model and evaluated the survival of patients through risk scores. The sample risk score formula is as follows:

(1) $$\begin{array}{c} \text{risk score}=\beta_{1}*{\text{Exp}}_{1}+\beta_{2}{\text{Exp}}_{2}+,\ldots ,\beta_{i}{\text{Exp}}_{i}. \end{array}$$

Among them, *β* was the value of the risk coefficient, and Exp represented the value of an expression in a certain gene. In accordance with the median value of risk score, NB patients were divided into two groups: low-risk group and high-risk group, and the survival difference between the two subgroups was compared through survival analysis. In addition, the prognostic ability of the above model is estimated through receiver operating characteristic curve (ROC) analysis. A sample of 276 NB patients with dependable follow-up information from the GSE85047 dataset was used as a validation group to evaluate the predictive power of the prognostic model. *P* < 0.05 was considered a statistically significant difference.

### 2.5. Validation of Hub Genes

For the selected hub genes, Kaplan–Meier estimated the survival differences between the low-risk and high-risk groups and validated in the GEO database (GSE85047), which is a publicly accessible online microarray database to facilitate discovery and identification of genome-wide expression analyses [11].

## 3. Results

### 3.1. Construction of Coexpression Modules

**Figure 1:**
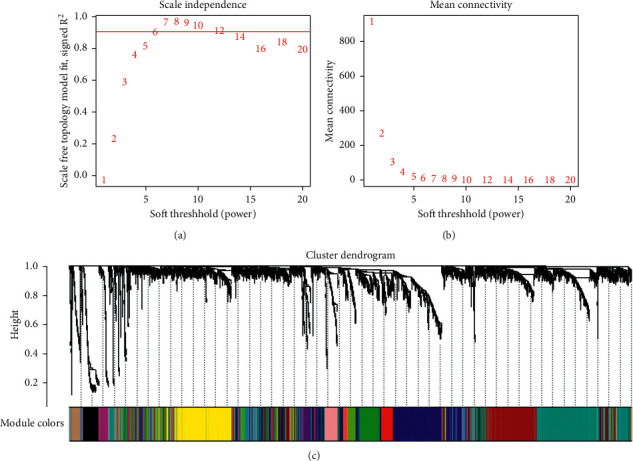
Determination of soft threshold (*β*) of weighted gene coexpression network analysis (a, b) and network module construction (c).

**Figure 2:**
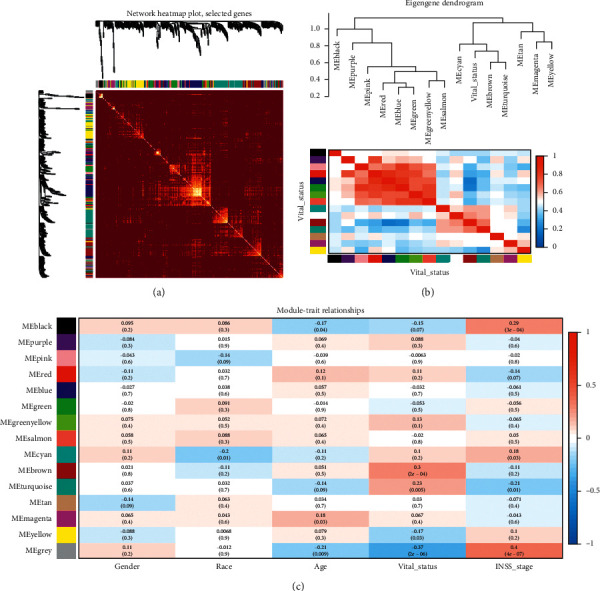
Identification of neuroblastoma and related modules with different clinical features. (a) WGCNA correlation clustering analysis of all genes. (b, c) Heat map correlation between different clinical features and module eigenvalues.

The raw data of neuroblastoma were downloaded from the TARGET database and contained expression values of 20,098 genes from 147 patients. The details of clinical/pathological features are listed in Table 1. The raw data are preprocessed by using R for background correction and normalization. Gene annotations are performed to match probes and gene symbols, and probes that match several genes are removed, and for genes matched by multiple probes, the median is considered the final expression value. The SD of each gene was calculated and ranged from large to small, and finally, 5,025 (top 25%) genes were selected for WGCNA analysis. First, the power value is filtered. When the power value is equal to 6, the degree of independence reaches 0.9, and the average connectivity is higher. Thus, the efficacy values and results used to construct the coexpression module revealed the identification of 14 different gene coexpression modules in neuroblastoma. These coexpression modules were constructed and displayed in different colors. These modules range from large to small, including their number of genes (Figures 1 and 2).

### 3.2. Coexpression Modules

**Figure 3:**
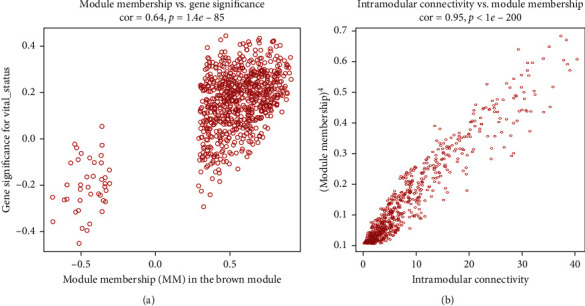
Scatter plot of module gene module membership and gene significance.

The interaction relationship of the modules is analyzed, and the network heat map is drawn. The results show that each module is independent of each other, demonstrating the high degree of independence between the modules and the relative independence of gene expression in each module. In addition, we calculated eigengenes and clustered them according to their correlation in order to explore the coexpression similarity of all modules, and similar results were represented by the heat maps drawn from the adjacency graphs. It is clear that the ME of the brown module showed a high correlation with the vital status compared to the other modules, with correlation coefficients of 0.3, *P* < 0.01. The brown module was positively correlated with the vital status, suggesting that the brown module may play an important role in neuroblastoma. We identified the brown module as the modules most relevant to the neuroblastoma's state (Figure 3).

### 3.3. Function Enrichment Analysis

**Figure 4:**
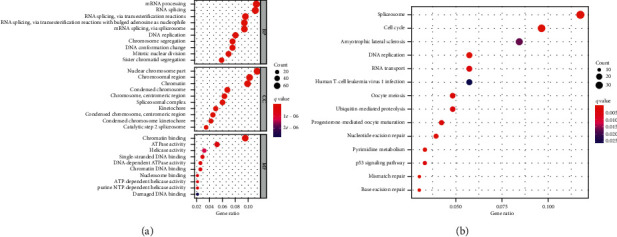
GO enrichment analysis and KEGG pathway analysis in the brown module.

We performed enrichment analysis to explore the GO and pathway in which the two key modules were involved. GO enrichment and the detailed information are given in Table 2. The result of functional enrichment analysis showed in brown module that in the biology processes that genes was mainly enriched in GO: 0006397 mRNA processing, GO: 0008380 RNA splicing, GO: 0034660 ncRNA metabolic process, GO: 0090501 RNA phosphodiester bond hydrolysis, GO: 0000377 RNA splicing, via transesterification reactions with bulged adenosine as nucleophile, GO: 0000398 mRNA splicing, via spliceosome, GO: 0000375 RNA splicing, via transesterification reactions, GO: 0090305 nucleic acid phosphodiester bond hydrolysis, GO: 0006401 RNA catabolic process, GO: 0034470 ncRNA processing, in the molecular function that in GO: 0140098 catalytic activity, acting on RNA, GO: 0004540 ribonuclease activity, GO: 0004518 nuclease activity, GO: 0003730 mRNA 3'-UTR binding, GO: 0004519 endonuclease activity, GO: 0045182 translation regulator activity, GO: 0140101 catalytic activity, acting on a tRNA, GO: 0003729 mRNA binding, GO: 0003725 double-stranded RNA binding, GO: 0004521 endoribonuclease activity, and in the cellular component in GO: 0035770 ribonucleoprotein granule, GO: 0036464 cytoplasmic ribonucleoprotein granule, GO: 0005840 ribosome, GO: 0044391 ribosomal subunit, GO: 0000313 organellar ribosome, GO: 0005761 mitochondrial ribosome, GO: 0000932 P-body, GO: 0005759 mitochondrial matrix, GO: 0043186 P granule, GO: 0045495 pole plasm. According to the Kyoto Encyclopedia of Genes and Genomes (KEGG) pathway analysis, our results demonstrated that these genes were mainly involved in mRNA surveillance pathway, RNA transport, Ribosome biogenesis in eukaryotes, RNA degradation, Ribosome, Aminoacyl-tRNA biosynthesis, Spliceosome, RNA polymerase. These results indicated that the clinical significant module genes were mainly involved in intracellular protein synthesis and were responsible for completing the process from RNA to protein in the “genetic central dogma” (Figure 4).

### 3.4. Identification of Hub Genes in the Module

**Figure 5:**
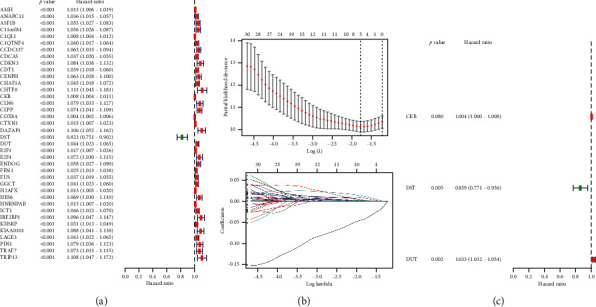
(a) Survival prognosis-related genes identified by univariate Cox analysis. (b) The result of Lasso analysis. (c) Survival prognosis-related genes identified by multivariate Cox analysis.

**Figure 6:**
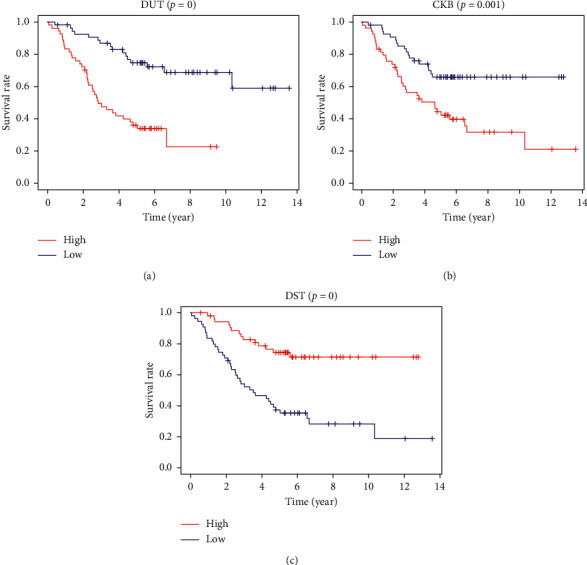
Effect of various hub genes of CKB, DST, and DUT on survival time of neuroblastoma.

The intramodule connectivity of each gene is calculated by adding the intensity of the linkage to other modular genes and dividing the number by the largest molar linkage. By calculating the correlation matrix between traits and genes, genes that are highly correlated with traits are also shown and are also key genes in the model associated with traits. According to this degree, genes with a high degree among the 14 clinically significant modules were identified as central genes. These genes with significant survival analysis results were selected and classified by node degree. Univariate Cox regression analysis was performed on these nodal genes and 37 candidate center genes related to prognosis. Subsequently, through Lasso regression, the prognostic risk equation of multifactor Cox regression was established (Figure 5). At last, CKB, DST, and DUT were identified as the key prognostic genes by the multivariate Cox regression analysis. We used these three hub genes to construct the predictive model (Figure 6).

The risk score of every child was calculated in accordance with the following formula:

(2) $$\begin{array}{c} \text{risk score}=\left(0.003812659*\text{CKB}\right)+\left(-0.152376975*\text{expDST}\right)+\left(0.032032815*\text{expDUT}\right). \end{array}$$

**Figure 7:**
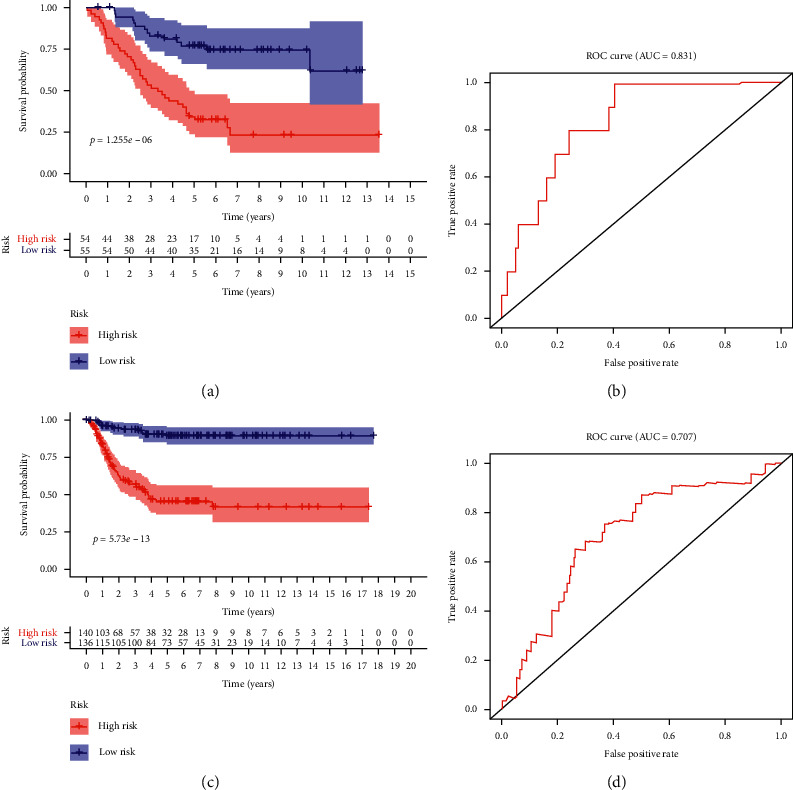
Relationship between model and survival in hub genes (a, b) and comparison of the GSE85047 database (c, d).

Then, based on the median value of risk scores, NB patients were divided into two groups: low-risk group and high-risk group. The results showed that compared with patients in the low-risk group, patients in the high-risk group had poorer survival, which was statistically significant (*P*=1.225*e* − 06). The value of the area under the curve (AUC) in the TARGET model is 0.831 (Figures 7(a) and 7(b)).

### 3.5. Validation of Hub Genes

**Figure 8:**
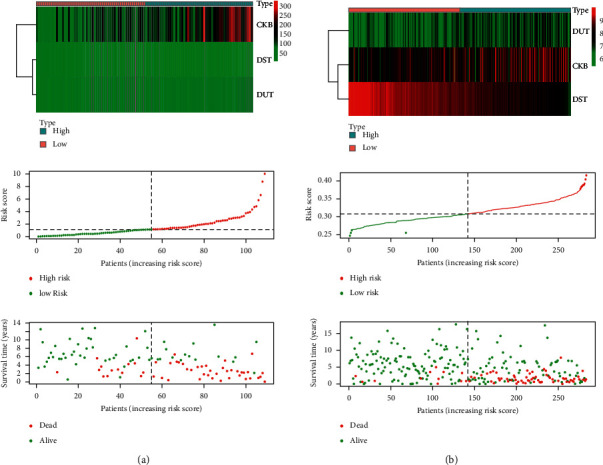
Risk score analysis of the 3-gene prognostic model. (a) TARGET cohort. (b) GSE85047 cohort.

With the purpose of evaluating of the prognostic value of the prediction model, we used the GSE85047 patient cohort to verify the relationship between risk score and survival time. In the GSE85047 cohort, groups were also grouped based on the median value of risk score in the TARGET model. The survival time of patients with high-risk scores was poorer for patients with lower risk scores, which was significant (*P*=5.73*e* − 13), and the AUC was 0.707 (Figures 7(c) and 7(d)). The distribution of risk scores and survival status revealed that gene expression increased relative to the rise of risk scores (Figure 8). In summary, these findings imply that the model has a good performance in predicting OS in NB patients.

### 3.6. Hub Genes' Model as an Independent Prognostic Factor

**Figure 9:**
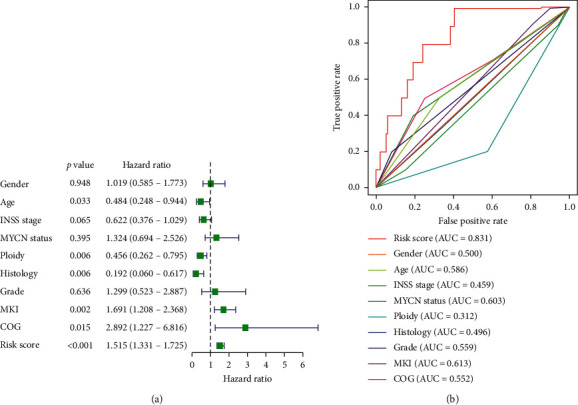
Univariate Cox regression analysis and receiving operating characteristic curve (ROC) of the correlation between the 3-gene signature risk score and clinical features in TARGET.

**Figure 10:**
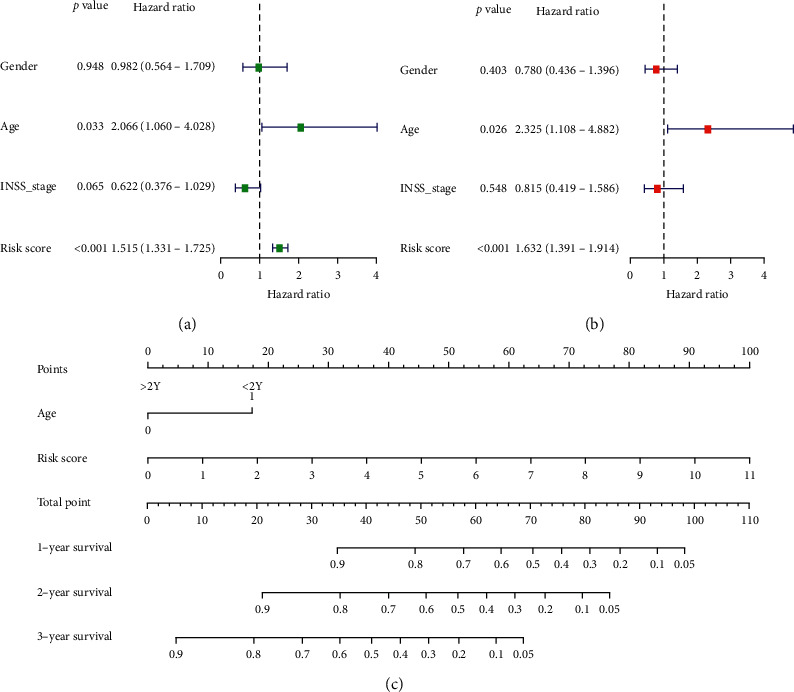
The nomogram can predict the prognosis probability in NB. (a) Univariate Cox regression analysis. Forest plot of associations between risk factors and the survival of NB. (b) Multiple Cox regression analysis. The RBP gene signature is an independent predictor of NB. (c) A nomogram of the NB cohort used to predict the OS.

We assessed the prognostic value of the risk scores of the model. For NB, the risk score in univariate analysis was significantly correlated with overall survival (OS) (HR = 1.515, 95% CI = 1.331–1.725, *P* < 0.001). ROC curve shows the prognostic value of the risk scores was better than Gender, Age, INSS Stage, MYCN status, Ploidy, Histology, Grade, MKI, and COG (Figure 9). Multivariate analysis showed that the risk score was an independent prognostic indicator (HR = 1.632, 95% CI = 1.391–1.934, *P* < 0.001). Nomogram integrates multiple risk factors to quantify individual risks in the clinical environment. We conducted a nomogram to predict the probability of OS in 1, 2, and 3 years (Figure 10).

## 4. Discussion

Neuroblastoma has become the second leading cause of death in children with malignant tumors. At present, the treatment of high-risk neuroblastoma mainly includes surgery, radiotherapy, and chemotherapy, as well as hematopoietic stem cell transplantation, but some patients have recurrence and metastasis during the treatment, leading to treatment failure. How to improve the cure rate and survival rate of these children is an urgent problem to be solved. [12].

The development and progression of neuroblastoma were complex and involved multiple molecules and pathways. Traditional research on only one or a few molecules cannot fully explore it. In recent years, with the development of high-throughput gene sequencing technology, researchers have obtained a large amount of genomics data and published it. However, this high-expression multiomics data also places higher demands on comprehensive data analysis—development mechanism. As a complex gene coexpression network construction method, WGCNA has unique advantages in dealing with multisample complex data [13]. WGCNA compensates for the shortcomings of traditional methods by identifying modules of functionally related genes in high-throughput data and considering gene function and its overall association with biological functions. Through this method of analysis, researchers can not only discover the relationship between genes within the module but also the relationship between the module and the genes in other modules. In addition, by correlating clinical information with modules, genes associated with clinical features can be further obtained, helping to lay the groundwork for studying the clinical features of the disease.

In this study, we applied the systematic biology method WGCNA to study the neuroblastoma gene expression dataset, and we identified 14 key gene coexpression modules. Among them, the brown gene module is closely related to vital status (cor = 0.3, *P* < 0.01). Functional enrichment analysis indicates that the module is highly correlated with gene pathway including mRNA processing, RNA splicing, ncRNA metabolic process, RNA phosphodiester bond hydrolysis, RNA splicing, via transesterification reactions with bulged adenosine as nucleophile, mRNA splicing, via spliceosome, RNA splicing, via transesterification reactions, nucleic acid phosphodiester bond hydrolysis, RNA catabolic process, ncRNA processing, suggesting an important role of these pathways in the pathogenesis of neuroblastoma. Here, our results indicate that these 3 genes may be new therapeutic gene targets for neuroblastoma.

Creatine kinase (creatine kinase, CK) is a very important class of kinases, which can precisely regulate the energy balance in the body and maintain the stability of intracellular ATP levels [14]. CKB is a subtype of creatine kinase, which is widely present in skeletal muscle, myocardium, nerve tissue, and mitochondria. It participates in the process of energy signal transduction, reversibly catalyzing the high-energy phosphate bond of phosphocreatine to combine with ADP to generate ATP and creatine [15]. CKB is one of the mechanisms of intrahepatic metastasis of colorectal cancer. Gastrin-release Pepper (GRP) and its receptor (GRPR) are abnormally highly expressed in colorectal cancer, while CKB is significantly higher in cell lines expressing GRP/GRPR [16]. CKB can shorten the survival period of tumor cells. In the microenvironment of liver cell hypoxia, CKB is regulated by miR-483 and miR-551a. It catalyzes the conversion of phosphocreatine to ATP, thereby providing energy for tumor cells that spread to the liver [17]. CKB is significantly abnormally expressed in tumor tissues. It has been studied in lung squamous cell carcinoma, prostate cancer, ovarian cancer, kidney cancer, and glioma and can be used as a new tumor marker for early diagnosis. [18].

DST was a member of the plakin protein family, which encodes adhesion junction plaque proteins. Mice with a defect in this gene show skin blistering and neurodegeneration. Dystonin plays an important role in tumor growth and angiogenesis of melanoma [19]. DST mutation is closely related to carcinoma of the oral tongue [20].

DUT encodes the basic enzyme of nucleotide metabolism and hydrolyzes dUTP into dUMP and pyrophosphate. Elevated dUTP levels induce extensive excision repair mediated by uracil glycosylase, which leads to DNA fragmentation and cell death. Deoxyuridine triphosphatase (dUTPase) has emerged as a potential target for drug development [21]. Deoxyuridine triphosphatase inhibitor has achieved significant clinical effects in many clinics and is currently a promising tumor-targeted therapy drug [22, 23].

Therefore, it can be inferred that these three genes are indeed the central genes responsible for the key process of NB, and they deserve more in-depth analysis and verification. Finally, by using machine learning methods, it is proved that the hub genes can effectively distinguish between NB samples and normal samples. In our study, the predictive effect of this method was evaluated by the AUC value. Here, the AUC value>0.8 indicates an excellent prediction result and is better than the INRG classification system such as INSS Stage, MYCN status, Ploidy, Histology, Grade, MKI, and COG, although Histology, Grade, MKI, and COG are independent prognostic factors. In addition, these three-gene models may be specific predictors of NB. All the results show that when distinguishing NB samples from normal samples, the expression profiles of these three central genes have an excellent predictive effect.

The development of targeted therapies has brought the dawn of treatment for children with high-risk NB. However, the safety and efficacy of targeted drugs still require more clinical trials to confirm. In addition, targeted drugs also have problems such as certain resistance and high prices. Therefore, the path of neuroblastoma targeted therapy is still full of challenges, but we believe that as the research on the mechanism of tumor development continues to deepen, more new effective targets will be discovered [24]. These findings may be translated into clinical practice in the near future, providing an effective means to improve the survival and quality of life in high-risk NB children.

In conclusion, this study proposes a functional organization of WGCNA on neuroblastoma transcriptomes. System-level view of gene expression profiles reveals coexpression modules associated with and reveals that the process of gene pathway systems involved in nervous system development, protein synthesis and that may be the pathogenesis of neuroblastoma plays an important role. Our findings provide new insights into the basis of genomics for neuroblastoma and provide potential therapeutic targets for precision medicine.

## Figures and Tables

**Table 1:** Clinical characteristics of NB patients in TARGET.

Characteristics	Number of cases (%)
Gender = male	87 (59.2)
Age (days) (mean (SD))	1281.51 (1098.47)

Vital status	
Alive	75 (51.0)
Dead	72 (49.0)

Overall survival time in days (mean (SD))	1730.50 (1120.27)

INSS stage	
Stage 1	0 (0.0)
Stage 2a	0 (0.0)
Stage 2b	1 (0.7)
Stage 3	6 (4.1)
Stage 4	119 (81.0)
Stage 4s	21 (14.3)

MYCN status	
Amplified	30 (20.4)
Not amplified	117 (79.6)

Ploidy	
Diploid (DI = 1)	63 (42.9)
Hyperdiploid (DI > 1)	84 (57.1)

Histology	
Favorable	28 (19.0)
Unfavorable	110 (74.8)
Unknown	9 (6.1)

Grade	
Differentiating	10 (6.8)
Undifferentiated or poorly differentiated	115 (78.2)
Unknown	22 (15.0)

MKI	
High	32 (21.8)
Intermediate	40 (27.2)
Low	47 (32.0)
Unknown	28 (19.0)

COG risk group	
High risk	120 (81.6)
Intermediate risk	13 (8.8)
Low risk	14 (9.5)

**Table 2:** Functional enrichment of genes in brown coexpression modules.

Ontology	ID	Description	*P*. adjusted	Count
BP	GO: 0006397	mRNA processing	4.74*E *−* *44	72
BP	GO: 0008380	RNA splicing	1.00*E *−* *33	59
BP	GO: 0034660	ncRNA metabolic process	2.81*E *−* *31	58
BP	GO: 0090501	RNA phosphodiester bond hydrolysis	2.81*E *−* *31	36
BP	GO: 0000377	RNA splicing, via transesterification reactions with bulged adenosine as nucleophile	5.58*E *−* *24	44
BP	GO: 0000398	mRNA splicing, via spliceosome	5.58*E *−* *24	44
BP	GO: 0000375	RNA splicing, via transesterification reactions	7.78*E *−* *24	44
BP	GO: 0090305	Nucleic acid phosphodiester bond hydrolysis	3.29*E *−* *22	38
BP	GO: 0006401	RNA catabolic process	5.85*E *−* *22	43
BP	GO: 0034470	ncRNA processing	6.99*E *−* *21	38
CC	GO: 0035770	Ribonucleoprotein granule	1.85*E *−* *21	32
CC	GO: 0036464	Cytoplasmic ribonucleoprotein granule	3.32*E *−* *19	29
CC	GO: 0005840	Ribosome	4.00*E *−* *13	27
CC	GO: 0044391	Ribosomal subunit	3.19*E *−* *12	22
CC	GO: 0000313	Organellar ribosome	1.05*E *−* *11	16
CC	GO: 0005761	Mitochondrial ribosome	1.05*E *−* *11	16
CC	GO: 0000932	P-body	3.01*E *−* *11	14
CC	GO: 0005759	Mitochondrial matrix	1.94*E *−* *10	31
CC	GO: 0043186	P granule	1.58*E *−* *08	7
CC	GO: 0045495	Pole plasm	1.58*E *−* *08	7
MF	GO: 0140098	Catalytic activity, acting on RNA	2.44*E *−* *27	45
MF	GO: 0004540	Ribonuclease activity	2.04*E *−* *17	23
MF	GO: 0004518	Nuclease activity	1.40*E *−* *16	28
MF	GO: 0003730	mRNA 3'-UTR binding	3.19*E *−* *13	17
MF	GO: 0004519	Endonuclease activity	3.73*E *−* *13	20
MF	GO: 0045182	Translation regulator activity	6.87*E *−* *12	14
MF	GO: 0140101	Catalytic activity, acting on a tRNA	1.73*E *−* *10	17
MF	GO: 0003729	mRNA binding	2.02*E *−* *10	33
MF	GO: 0003725	Double-stranded RNA binding	2.18*E*−10	14
MF	GO: 0004521	Endoribonuclease activity	2.76*E *−* *10	13

## Data Availability

The datasets used to support the findings of this study are available in the Therapeutically Applicable Research to Generate Effective Treatments project database (TARGET, https://ocg.cancer.gov/programs/target) and Gene Expression Omnibus (GEO) (https://www.ncbi.nlm.nih.gov/geo/).
